# The Potential Role of Activating the ATP-Sensitive Potassium Channel in the Treatment of Hyperphagic Obesity

**DOI:** 10.3390/genes11040450

**Published:** 2020-04-21

**Authors:** Neil Cowen, Anish Bhatnagar

**Affiliations:** Soleno Therapeutics, Redwood City, CA 94065, USA; anish@soleno.life

**Keywords:** K_ATP_ channel activation, hyperphagic obesity, animal models, Prader–Willi syndrome

## Abstract

To evaluate the potential role of ATP-sensitive potassium (K_ATP_) channel activation in the treatment of hyperphagic obesity, a PubMed search was conducted focused on the expression of genes encoding the K_ATP_ channel, the response to activating the K_ATP_ channel in tissues regulating appetite and the establishment and maintenance of obesity, the evaluation of K_ATP_ activators in obese hyperphagic animal models, and clinical studies on syndromic obesity. K_ATP_ channel activation is mechanistically involved in the regulation of appetite in the arcuate nucleus; the regulation of hyperinsulinemia, glycemic control, appetite and satiety in the dorsal motor nucleus of vagus; insulin secretion by β-cells; and the synthesis and β-oxidation of fatty acids in adipocytes. K_ATP_ channel activators have been evaluated in hyperphagic obese animal models and were shown to reduce hyperphagia, induce fat loss and weight loss in older animals, reduce the accumulation of excess body fat in growing animals, reduce circulating and hepatic lipids, and improve glycemic control. Recent experience with a K_ATP_ channel activator in Prader–Willi syndrome is consistent with the therapeutic responses observed in animal models. K_ATP_ channel activation, given the breadth of impact and animal model and clinical results, is a viable target in hyperphagic obesity.

## 1. Introduction

Hyperphagic obesity is characterized by a markedly increased appetite and aggressive food-seeking behavior, often associated with a lack of satiety, and the accumulation of excess body fat, frequently resulting in morbid obesity and obesity-associated comorbidities. The underlying basis for hyperphagic obesity is frequently genetic, involving biallelic-inactivating mutations in known genes, as is the case of leptin receptor deficiency [1], or the deletion or lack of expression of a chromosomal segment containing a number of genes, which is characteristic of Prader–Willi syndrome [2]. Alternatively, hyperphagic obesity may follow from damage to the hypothalamus, resulting in hypothalamic obesity [3]. Hyperphagic obesity is frequently associated with both elevated morbidity and mortality and reduced quality of life [2,3]. There are very few approved treatments for any form of hyperphagic obesity and, therefore, there is a need to identify effective therapeutic targets to address the unmet medical need in these conditions. This review focuses on a single potential therapeutic target, the ATP-sensitive potassium (K_ATP_) channel, which may have utility in various forms of hyperphagic obesity.

## 2. Methods

A PubMed search was conducted focused on the expression of genes encoding the K_ATP_ channel in tissues involved in the regulation of appetite and satiety, and in tissues involved in the establishment and persistence of the obese state, the role of activating the K_ATP_ channel in those tissues, studies involving pharmacological activators of the channel in obese hyperphagic animal models, and clinical studies of K_ATP_ channel activators in obese hyperphagic syndromes. Search terms used included K_ATP_, SUR1, SUR2b, Kir6.1, Kir6.2, ABCC8, ABCC9, KCNJ8, KCNJ11, agonist, hypothalamus, motor neuron of vagus, adipocyte, β-cell, hyperphagia, appetite, neuropeptide, obesity, obese, animal model, leptin, insulin, α-MSH, insulin-resistance, and hyperinsulinemia. Terms were combined to generate searches which identified tissues in which the genes encoding the K_ATP_ channel might be expressed that have a known role in appetite and obesity, hormones with known roles in appetite and the K_ATP_ channel, and obese or hyperphagic obese animal models in which a K_ATP_ channel agonist might have been evaluated. Prior to conducting the searches, the authors already possessed extensive knowledge of the K_ATP_ channel and its role in the regulation of appetite, having studied the channel for more than 15 years. The searches were conducted to supplement that understanding, rather than as the sole source of information summarized in this publication.

## 3. Results

### 3.1. The Structure of and Genes Encoding the K_ATP_ Channel

The K_ATP_ channel is an octomeric structure consisting of four copies of the sulfonylurea receptor (SURx) and four copies of an inwardly rectifying potassium channel (Kir6.x) [4]. The isotype of the channel is a function of the specific forms of each of the two components that are expressed in the tissue at any developmental stage. SUR1 is encoded by ABCC8; SUR2A and SUR2B are splice variants of the same gene product which is encoded by ABCC9 [4]. Kir6.2 is encoded by KCNJ11, while Kir6.1 is encoded by KCNJ8 [4]. ABCC8 and KCNJ11 both reside on chromosome 11p15.1, while ABCC9 and KCNJ8 both reside on chromosome 12p12.1 [4]. SUR1-containing forms of the channel are typically found in pancreatic β-cells, certain CNS cell types [5], adipocytes [6] and certain skeletal muscle cells but exist in other tissues as well. SUR2B-containing forms of the channel are typically found in cardiovascular smooth muscle [4] and certain skeletal muscle cells, whereas SUR2A-containing forms of the channel are found almost exclusively in cardiac tissues [4]. The channel resides in the cell membrane, and a unique isotype resides in the mitochondrial inner membrane [4].

### 3.2. The Role of the K_ATP_ Channel in the Central Regulation of Appetite by the Arcuate Nucleus

In mature animals or humans, the arcuate nucleus of the hypothalamus (ARC) integrates peripheral circulating hormonal signals, including leptin, insulin and ghrelin, which communicate environmental nutrient availability, into the homeostatic regulation of appetite and energy expenditure. The ARC, in the mature animal, includes two populations of neurons that respond to these hormonal signals and project into other appetite-regulatory regions. One population of neurons (NAG neurons) co-expresses neuropeptide Y (NPY), the most potent endogenous appetite-stimulatory neuropeptide, agouti-related protein (AgRP), an antagonist/inverse agonist of melanocortin 4 receptors (MC4R) [7], and γ-aminobutyric acid (GABA). NAG neurons are orexigenic and sufficient to regulate food intake [5]. A separate population of ARC neurons (POMC neurons) expresses the proopiomelanocortin (POMC) peptide and are anorexigenic. Within the ARC, POMC neurons are inhibited by NAG neurons [8], mediated primarily by GABA. Additionally, NPY has been shown to downregulate prohormone convertase 2 [9], which is a key enzyme involved in the conversion of POMC to α-melanocyte-stimulating hormone (α-MSH) which, through its interaction with MC4R, mediates the anorexigenic effects of POMC neurons. Thus, NAG neurons serve a modulatory function, through direct inhibition of POMC neurons, reduced conversion of POMC to α-MSH, and antagonism of MC4R, thereby counter-regulating the melanocortin pathway to reduce satiety and promote food intake. During early postnatal development, projections from these two populations of neurons extend to other key appetite-regulatory regions including the paraventricular nucleus (PVH), the dorsomedial nucleus (DMH), and lateral hypothalamic area (LHA), exerting opposing effects on appetite and energy expenditure [10].

NPY injected into the brain either in the ventricles or in different hypothalamic nuclei induces a robust feeding response, even in sated animals [11]. NPY achieves this effect by reducing the latency to eat, delaying satiety and thereby augmenting meal size and meal duration [11]. NPY also causes treated animals to be more motivated to obtain food [11]. Specifically activating neurons pharmacologically with AgRP induces a robust hyperphagic response in rodents with a distinct temporal dynamic from that of NPY [7], NPY results in immediate feeding while AgRP increases food intake, but the effect is delayed and occurs over a longer time scale. In adult animals, NAG neuron ablation results in rapid starvation [12].

**Figure 1:**
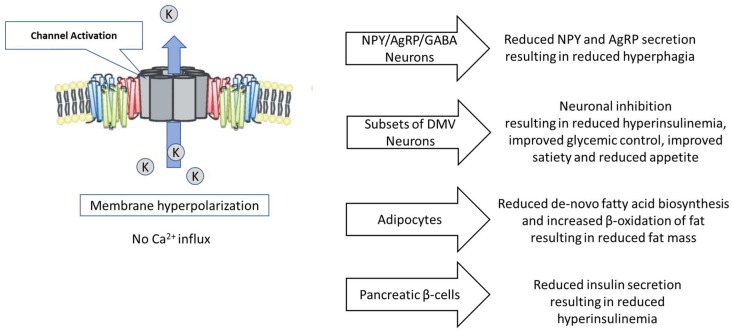
The Potential Role of the K_ATP_ Channel in the Regulation of Cellular and Physiological Processes Associated with Establishing and Maintaining the Obese Hyperphagic State.

Leptin inhibits the excitability of NAG neurons, reducing their firing rate and hyperpolarizing their resting membrane potential [13]. This inhibition is mediated through leptin’s activation of ATP-sensitive potassium channels (K_ATP_) via phosphoinositide-3-kinase (PI3-K) [5,14,15]. Activation of K_ATP_ channels (i.e., keeping them open) serves to hyperpolarize the resting membrane potential. While it is unclear whether hyperpolarizing the resting membrane potential affects intra-cellular proNPY mRNA or NPY protein levels, it clearly results in limiting the release of NPY by these neurons (Figure 1). Since NPY and AgRP are co-localized in the same secretory vesicles, factors that affect NPY secretion affect AgRP secretion to the same degree [16]. Depolarizing the NAG neuron resting membrane potential results in a doubling of the NPY release rate, which returned to normal values when the resting membrane potential returned to a neutral condition [17]. In adult animals, K_ATP_ channels in the NAG neurons appear to include only SUR1, but not SUR2 [5,18]. At this stage, exposure to diazoxide free base (DFB), a potent K_ATP_ channel activator, resulted in significant hyperpolarization of the resting membrane potential in 100% of NAG neurons, and DFB more extensively hyperpolarized the resting energy potential of NAG neurons than leptin [5].

### 3.3. The Role of the K_ATP_ Channel in the Regulation of Neuronal Function in the Dorsal Motor Nucleus of the Vagus

The dorsal vagal complex (DVC) is an autonomic regulatory center located in the caudal medulla. Primary viscerosensory information is processed within the nucleus tractus solitarii (NTS) and subsequently relayed to the dorsal motor nucleus of vagus (DMV). Neurons within the DMV are parasympathetic motor neurons as they project to the periphery and regulate the tone of most of the subdiaphragmatic organs and, thus, regulate feeding, digestion, and energy and glucose homeostasis [19,20]. The activity of DMV neurons is largely controlled by local circuits and by inputs from other brain regions including the hypothalamus [21,22,23]. Hormones, metabolic signals, gastrointestinal signals, or pharmacological agents have the potential to alter the activity of DMV neurons and thereby modulate the parasympathetic outflow to the organs.

Deletion of the melanocortin 4 receptor (MC4R) in the DMV results in hyperinsulinemia and modest insulin resistance in a weight-independent manner and without changes in glucose tolerance or blood glucose levels [24]. Sohn et al. [25] examined the role of melanocortin 4 receptors (MC4R) in the regulation of cholinergic neurons of the DMV which have been hypothesized to regulate insulin secretion. In these studies, MTII (a MC3R/MC4R agonist) hyperpolarized the membrane potential of cholinergic neurons within the DMV, but failed to hyperpolarize the membrane potential of cholinergic neurons in mice which were deficient for MC4R. The MTII-induced hyperpolarization of DMV cholinergic neurons was accompanied by a decrease in input resistance characteristic of the activation of a K^+^ channel which was reversed by tolbutamide, a K_ATP_ antagonist, leading to the conclusion that the K^+^ channel mediating MC4R-induced membrane hyperpolarization in these neurons is the K_ATP_ channel. The results of this study show that activation of MC4Rs decreases parasympathetic tone following the activation of K_ATP_ channels, which may result in decreased insulin secretion.

Insulin-induced hypoglycemia enhances vagal activity [26], while hyperglycemia depresses vagal tone [27]. Eliminating or blocking components of the insulin receptor pathway centrally, such as phosphatidylinositol 3 kinase (PI3K) or the K_ATP_ channel, disrupts vagal control of energy homeostasis [28,29], and insulin applied centrally improves hepatic gluconeogenesis in a vagally mediated manner in models of diabetes [30,31,32]. Moreover, insulin applied to the dorsal vagal complex affects the function of peripheral targets [33,34]. Thus, insulin may alter neural activity in the dorsal vagal complex to affect visceral function. Blake et al. [35] evaluated the effect of insulin on excitation of gastric-related neurons in the DMVand showed that insulin acts on insulin receptors located on glutamatergic afferent terminals synapsing on DMV neurons. They also showed that insulin did not affect inhibitory transmission in DMV neurons, and that there are direct effects of insulin on action potential (AP) frequency in DMV neurons, independent of fast synaptic transmission. They showed that the decrease in AP frequency was consistent with a PI3K-dependent activation of the K_ATP_ channel. Pocai et al. [36] showed that mice lacking the SUR1 subunit of the K_ATP_ channel are resistant to the inhibitory effect of insulin on gluconeogenesis, suggesting that SUR1 containing K_ATP_ channels are responsible for insulin-mediated actions within the dorsal motor nucleus of vagus.

Grill et al. [37] have reported leptin receptor gene expression in the dorsal vagal complex, including the DMV. In addition, fourth ventricle administration of leptin reduces food ingestion and weight gain, and these appetite effects are mimicked by microinjection of leptin into the DVC. Williams et al. [38] studied the effects of leptin on membrane potential in DMV neurons. They showed that leptin hyperpolarizes the majority of DMV neurons, which was consistent with the opening of a K^+^ channel. Using tolbutamide and a PI3K inhibitor, the authors showed that leptin failed to hyperpolarize DMV neurons pre-exposed to tolbutamide and the application of tolbutamide depolarized neurons that had been hyperpolarized by leptin, implicating the K_ATP_ channel in the hyperpolarization. Leptin receptors are expressed widely in autonomic centers implicated in regulating ingestive behaviors, including the vagal complex and several hypothalamic sites. DMV motor neuron activity has been linked with behaviors related to feeding and satiety [38]. Enhancement of feeding behavior is directly associated with increases in DMV motor activity, whereas satiety is directly correlated with decreases in DMV motor activity [39,40]. Leptin suppression of DMV motor activity may result in satiety [38]. Additionally, leptin suppression of activity within the DVC may be closely related to the autonomic effects of leptin on blood pressure, glucose production, and/or insulin sensitivity [41,42,43].

Leptin, insulin and α-MSH interacting with their respective receptors, LepR, InsR, and MC4R, each achieve some or all of their respective effects in the DMV via a PI3K-mediated opening of the K_ATP_ channel, resulting in membrane hyperpolarization which leads to inhibition of neuronal signaling. The use of a K_ATP_ channel activator which is capable of penetrating the DVC has the potential to recapitulate the effects of leptin, insulin and α-MSH, with the likely result being reductions in hyperinsulinemia, reductions in hepatic gluconeogenesis, reductions in appetite and improved satiety (Figure 1).

### 3.4. The Role of The K_ATP_ Channel in the Regulation of Adipocyte Metabolism and Fat Mass

There is also evidence that activation of K_ATP_ channels in peripheral tissues, including adipose tissue, may be important for body weight regulation. Shi et al. [6] detected the expression of SUR1 in human adipocytes using RT-PCR. Shi [6] evaluated lipogenesis in human adipocytes treated with a K_ATP_ channel activator or an antagonist, using fatty acid synthase (FAS), which catalyzes the synthesis of palmitate from acetyl-CoA and malonyl-CoA, and glycerol-3-phosphate dehydrogenase (GPDH) activities as lipogenic markers. Glibenclamide, a K_ATP_ channel antagonist, caused a significant increase in FAS and GPDH activity, which were completely blocked by DFB. Forty eight-hour treatment with glibenclamide caused a significant inhibition in lipolysis, which was substantially recovered by DFB exposure. Thus, closing the K_ATP_ channel in human adipocytes increased the de novo synthesis of fatty acids and downregulated lipolysis, while activating the channel substantially reversed these effects.

Alemzadeh et al. [44] evaluated the effect of activating the K_ATP_ channel on fat oxidation rate in obese Zucker rats. The Zucker rat has defective leptin signaling due to a mutation in the leptin receptor which results in hyperphagic obesity, in which reduced lipid oxidation contributes to the obese state. Obese Zucker rats were treated either with vehicle or DFB. Treated animals had significantly higher fat oxidation without a significant change in glucose oxidation, as well as lower fat mass compared to vehicle-treated animals. Alemzadeh and Tushaus [45] evaluated the effect of activating the K_ATP_ channel on the expression of genes involved in hepatic fatty acid biosynthesis in Zucker diabetic fatty (ZDF) rats. ZDF rats carry the same defect in leptin signaling as Zucker fatty rats and also have a genetic predisposition to diabetes. ZDF rats were randomized to DFB or vehicle control. Treatment was associated with mRNA reductions of hepatic sterol regulatory element-binding protein-1c, FAS, acetyl CoA carboxylase, hormone-sensitive lipase, and peroxisome proliferator agonist receptor-γ, without altering acyl-CoA oxidase, peroxisome proliferator receptor-α, or carnitine palmitoyl transferase-1. ACC and FAS are the enzymes primarily responsible for the synthesis of medium- and long-chain fatty acids, where ACC catalyzes the first synthetic step and FAS catalyzes a series of 2-carbon additions.

The K_ATP_ channel appears to be a key regulator of both fatty acid biosynthesis and β-oxidation of fat. One of the peripheral effects of treatment of obese individuals with a K_ATP_ channel activator should be loss of body fat (Figure 1). Similarly, K_ATP_ channel activators, by this mechanism, should prevent the accumulation of excess body fat in individuals who are prone or genetically predisposed to such accumulation.

### 3.5. The Role of the K_ATP_ Channel in Reducing Hyperinsulinemia

The K_ATP_ channel has a crucial role in insulin secretion which was summarized in the following way by Komatsu et al. [46]. On elevation of plasma glucose concentration, glucose enters the pancreatic β-cells through the glucose transporter on the plasma membrane. Glucose is then phosphorylated by glucokinase and subjected to glycolysis, by which pyruvate is generated in the cytoplasm. Pyruvate is metabolized equally by pyruvate dehydrogenase and pyruvate carboxylase (PC) in the β-cells and passes into the mitochondria. The former reaction leads to generation of adenosine triphosphate (ATP) in the respiratory chain and the latter is accompanied by efflux of tricarboxylic acid (TCA) cycle intermediates as anaplerosis. ATP is a signaling molecule for insulin secretion in β-cells, because the cell is equipped with ATP-sensitive K^+^ channels (K_ATP_ channels), which close on elevation of cytoplasmic ATP or ATP/adenosine diphosphate ratio. As the K_ATP_ channel is the primary determinant of the membrane potential of the β-cells, closure of these channels causes membrane depolarization. The membrane depolarization opens L-type voltage-dependent Ca^2+^ channels (VDCC), followed by Ca^2+^ influx and elevation of cytosolic free Ca^2+^ concentration ([Ca^2+^]_i_). The elevation of [Ca^2+^]_i_ rapidly increases the rate of insulin exocytosis.

Insulin is a powerful anabolic hormone secreted from pancreatic β-cells that acts on multiple tissues to stimulate the synthesis and storage of carbohydrates, lipids, and proteins [47]. Adipose tissue remodeling can occur via lipid hypertrophy and/or hyperplasia, and there is evidence that insulin has direct effects on cellular lipid metabolism [48] and adipocyte differentiation [49]. Insulin acts to inhibit lipolysis in adipocytes in the postprandial state [50], while promoting lipid storage through stimulating the uptake, synthesis, and storage of triglycerides in adipocytes [51]. Insulin stimulates adipogenesis via multiple mechanisms [52].

Hyperinsulinemia is a very early pathobiological event in the cascade of homeostatic dysfunction leading to obesity, insulin resistance, and type 2 diabetes [53]. For example, preclinical evidence from male C57BL/6 mice shows that insulin levels are elevated several weeks prior to the onset of obesity [54]. Similarly, hyperinsulinemia precedes insulin resistance, obesity, and enhanced lipogenesis in Lep ob/ob mice [55,56,57]. Onset of hyperinsulinemia is a primary causal factor in animal models of obesity and in some human populations [47]. Induction of VMH lesions in animal models results in hyperinsulinemia, obesity and hyperphagia [58]. The use of streptozotocin in these models results in the partial destruction of pancreatic β-cells, which reverses the hyperphagia and weight gain observed in the model [59]. This suggests that both hyperphagia and weight gain follow from hyperinsulinemia in lesioned animals. On the other hand, if there is strict control of energy intake in lesioned animals, there is no hyperphagia, but hyperinsulinemia and weight gain occur nonetheless [60,61].

K_ATP_ channel activators can partially suppress insulin secretion, are approved for the treatment of hyperinsulinemia, and would be useful to reduce the contribution of hyperinsulinemia to hyperphagia and obesity (Figure 1).

### 3.6. Insulin Resistance and Hyperinsulinemia

While it is clear that increases in insulin resistance can result in hyperinsulinemia, it is equally true that persistent hyperinsulinemia contributes to the development of insulin resistance both by reductions in the numbers of receptors and by postreceptor effects. Kobayashi and Olefsky [62] studied the effect of experimental hyperinsulinemia on insulin resistance in rat adipocytes. Hyperinsulinemia was induced using a gradually increasing dose of insulin, which led to a significant reduction in insulin receptors and a rightward shift in the insulin-glucose transport dose–response curve. The decrease in insulin sensitivity is the predicted functional consequence of these observed changes. When the insulin injections were stopped, insulin receptors and insulin sensitivity rapidly returned to normal. Hyperinsulinemia also increased insulin resistance in humans over 2 day treatment with exogenous insulin [63], and in patients with insulinomas [64].

Obici et al. [31] investigated the effect of downregulation of hypothalamic insulin receptor, as is characteristic of insulin resistance, on hyperphagia and fat mass and showed that the selective decrease in hypothalamic insulin receptor protein was accompanied by a rapid onset of hyperphagia and increased fat mass. Thus, central insulin resistance likely also contributes to hyperphagia and obesity.

Improvements in insulin sensitivity can be achieved using a K_ATP_ channel activator. Ratzmann et al. [65] studied the impact of K_ATP_ channel activator treatment on insulin sensitivity in obese subjects. Insulin sensitivity was studied on two separate days before and after 4 days of treatment with DFB. The authors concluded that treatment resulted in a 50% increase in insulin sensitivity in comparison to the pretreatment value. Wigand and Blackard [66] showed that 7 days of treatment with a K_ATP_ channel activator resulted in increased expression of both high and low affinity insulin receptors in treated subjects. In animal models, K_ATP_ channel activator-mediated improvements in insulin sensitivity were conditioned by increased mRNA and protein levels for glucose transporter 4 and increased protein expression for insulin receptor substrate-1 [67], and hepatic tissue levels of activated PKB/Akt (p-Akt, phosphorylated Akt), expressed as a ratio of p-Akt to Akt protein (p-Akt/Akt), were increased by treatment. DFB treatment of Zucker diabetic fatty rats resulted in improved insulin resistance compared to controls [68].

Škrha et al. [69] studied the effect of K_ATP_ channel activator treatment on insulin resistance in insulinoma and control subjects. A euglycemic clamp procedure was applied to subjects before and after 3 days of DFB administration. At baseline, the insulinoma patients were more insulin resistant than the controls, as measured by glucose disposal rate and metabolic clearance rate of insulin. Three days of treatment with DFB resulted in reduced basal insulin levels, increased tissue sensitivity to insulin and improved metabolic clearance rate of insulin. After treatment, the insulin sensitivity of insulinoma subjects was not significantly different from healthy control subjects.

### 3.7. The Use of K_ATP_ Channel Activators in Animal Models of Hyperphagic Obesity

The following animal models have been shown to be hyperphagic: Zucker fatty rat [70], Zucker diabetic fatty rat [70], db/db mouse [70], Otsuka Long Evans Tokushima Fatty rat [70], ventromedial hypothalamus lesioned animal models [70], and streptozotocin-induced diabetic rat [71]. The Magel2 mouse can be rendered obese with some elevation of appetite by ad libitum access to a high-fat diet [72]. The high-fat diet-induced obese (DIO) mouse also appears to have elevated appetite, while the DIO rat is clearly hyperphagic [70]. The experience with K_ATP_ channel activators in these nine models is discussed below. In animal models, changes in hyperphagia are typically assessed in the context of ad libitum access to food and measured directly by assessing either changes in energy intake or indirectly by the evaluation of the impact of changes in hyperphagia on weight and fat mass.

#### 3.7.1. Magel2 Knockout Mice—A Model of Prader–Willi Syndrome

Magel2, encoding a MAGE-like protein, resides within the Prader–Willi syndrome (PWS) critical region of chromosome 15 in humans and its loss or lack of expression may account for several of the observed characteristics of the disease. When administered a high-fat diet, Magel2 null mice display several features of PWS, including elevated appetite and obesity [73]. In a study by Bischof and Wevrick [72], wild-type and Magel-2 null mice were rendered obese after being fed a high-fat diet. Both sets of mice received DFB with continued ad libitum access to a high-fat diet. With treatment, both groups of mice showed significant weight loss, and fat mass loss. Fasting glucose measurements of both strains after 12 weeks of HFD were in the high normal range. Four weeks of treatment significantly lowered fasting glucose measurements of both strains. The Magel2 null mice were better able to sustain energy expenditure in treadmill tests following treatment compared to their pretreatment performance.

#### 3.7.2. Zucker Fatty Rat—A Model of LepR Deficiency

The Zucker fatty rat has a mutation in the leptin receptor which renders NAG neurons and other tissues non-responsive to leptin regulation. NPY messenger RNA levels are elevated 2- to 3-fold in Zucker fatty rats compared to their lean littermates [74]. This results in hyperphagia and obesity.

There are multiple publications covering the use of K_ATP_ channel activators in Zucker fatty rats [44,75,76,77,78,79]. The results of Alemzadeh and Holshouser [77] are provided by way of example. Eleven-week-old animals were randomized to receive DFB or vehicle control. Treatment was associated with a significant reduction in energy consumed per day per 100 gm body weight, reductions in plasma glucose and reductions in fasting insulin and leptin compared to vehicle-treated animals. Alemzadeh et al. [44] showed that relative to vehicle-treated controls, DFB treatment significantly reduced energy intake, weight gain, and circulating triglycerides and showed increased β-oxidation of fat and basal metabolic rate (BMR).

#### 3.7.3. Zucker Diabetic Fatty Rat—A Model of LepR Deficiency

The Zucker diabetic fatty rat includes a further mutation, which, in the context of the LepR mutation in this strain, accelerates the development of diabetes.

Alemzadeh and Tushaus [45,68] conducted two studies of the effect of activating the K_ATP_ channel in the Zucker diabetic fatty rat. In each study, six-week-old animals were subdivided into three groups, DFB treated, pair-fed and control animals, with the latter two receiving vehicle treatment. Treated animals gained significantly less weight than the pair-fed or control animals, even though the pair-fed animals consumed the same energy per day. Treated animals consumed significantly less energy per day compared to controls. Compared to either pair-fed or control animals, treated animals also had significantly lower glucose, higher insulin, lower triglycerides, lower HbA1c, and lower hepatic triglyceride and cholesterol content.

#### 3.7.4. db/db Mouse—A Model of LepR Deficiency

Both NPY and AgRP levels are elevated in db/db diabetic mice [80], contributing to hyperphagia. Lee [81] evaluated the effect of K_ATP_ channel activator treatment on food intake in (db/db) diabetic mice. Diabetic (db/db) mice consume nearly twice the amount of food as do control heterozygous mice. Treatment with DFB resulted in dose dependent reductions in food intake. At the highest dose, food intake was reduced by 50% compared to control diabetic animals.

#### 3.7.5. Otsuka Long Evans Tokushima Fatty Rat—A Model of CCK1 Receptor Deficiency

Hyperphagia in the Otsuka Long Evans Tokushima fatty (OLETF) rat is due to the absence of cholecystokinin (CCK)-1 receptors in both the gastrointestinal track and the brain. OLETF rats have a deficit in their ability to limit the size of meals and in contrast to CCK-1 receptor knockout mice, do not compensate for this increase in the size of their spontaneous meals, resulting in hyperphagia. Prior to becoming obese and in response to pair feeding, OLETF rats have increased expression of NPY in the compact region of the dorsomedial hypothalamus (DMH), and this overexpression contributes to their overall hyperphagia [82].

Guo et al. [83] evaluated the effects of treatment with a K_ATP_ channel activator in the OLETF rat. DFB or vehicle control was administered starting at 8 through 30 weeks of age. Long Evans Tokushima Otsuka (LETO) rats also served as controls. LETO rats are not genetically predisposed to obesity or diabetes. Weight gain over the 22 weeks in the treated OLETF animals was less than in the LETO controls and significantly less than in the OLETF vehicle-treated animals. Compared to controls, treated animals had significantly lower circulating triglyceride and less intra-abdominal fat, and markedly lower accumulation of fat in the liver and pancreas. Fasting glucose and insulin in treated animals were comparable to the non-diabetic LETO animals and significantly lower than controls.

#### 3.7.6. Hypothalamic Injured Rat—A Model of Hyperinsulinemia Driven Hyperphagic Obesity

In animals, ventromedial hypothalamic (VMH) lesions cause hyperphagia and obesity [61]. In these animals, there is central dysregulation of insulin secretion resulting in hyperinsulinemia, obesity and hyperphagia. Both hyperphagia and weight gain follow from hyperinsulinemia in this model. If there is restriction of energy intake in lesioned animals, there is no hyperphagia, but hyperinsulinemia and weight gain occur nonetheless.

Larue-Achagiotis and Le Magnen [84] evaluated the effect of K_ATP_ channel activator treatment on feeding behaviour in normal and VMH lesioned Wistar rats. DFB was administered as an intra-venous dose. Rats are typically nocturnal feeders. Meal size during the night is approximately 2x the average daytime meal size. Rats also eat more meals per night than during the day. Lesioned animals increased their food intake by more than 50% at night and by approximately 300% during the day, and tend to consume similar meal sizes in both daytime and night-time. Treatment reduced food intake in a dose dependent manner. At lower doses, treatment induced a latent period without a change in meal size. At higher doses, treatment was associated with a longer latency period followed by reduced meal size.

#### 3.7.7. Hypothalamic Injured Chicken—A Model of Hyperinsulinemia Driven Hyperphagic Obesity

The effect of activating the K_ATP_ channel on hyperphagia associated with hypothalamic injury in a white leghorn chicken model was evaluated by Sonoda [85]. Controls underwent a sham operation. Lesioned animals showed a 2-fold increase in food consumption. DFB was administered for 4 days. Treatment resulted in a statistically significant reduction in food consumption. Average daily food consumption increased once treatment was withdrawn.

#### 3.7.8. Streptozotocin Induced Diabetic Rat

NPY expression is also elevated in rats made diabetic by streptozotocin treatment, contributing to hyperphagia and weight gain in these models [71]. Matsuda et al. [86] evaluated the effects of activating the K_ATP_ channel in a streptozotocin-induced diabetic Wistar rat model. Male Wistar rats were either treated with a 30 mg injection of streptozotocin (to induce diabetes) or vehicle injection. One week following streptozotocin treatment, animals were randomized to either DFB or vehicle. The authors did not evaluate food consumption, but they did examine rate of weight gain at 2, 4 and 6 weeks. At each time point, treated animals showed significantly lower rates of weight gain than vehicle control animals.

#### 3.7.9. High-Fat Diet-Induced Obese Mouse

The effect of activating the K_ATP_ channel in high-fat diet-induced obese mice was evaluated by Bischof and Wevrick [72] and described above in relation to the Magel2 mouse model of PWS. Surwit [87] also evaluated the effect of activating the K_ATP_ channel on high-fat diet-induced obese mice. After 4 weeks on the high-fat diet (HFD), mice were randomized to control or DFB treatment groups and continued HFD. Compared to HFD controls, treated HFD animals showed a significantly lower rate of weight gain, lower leptin, lower circulating non-esterified fatty acids and triglycerides, lower epididymal and retroperitoneal fat pad weights, lower plasma insulin, and lower plasma glucose. Glucose and insulin levels in treated HFD animals were markedly lower than LFD controls.

#### 3.7.10. Tabular Summary of Animal Model Results

A tabular summary of responses to activating the K_ATP_ channel in nine animal models of hyperphagic obesity is presented in Table 1.

### 3.8. Experience with the Diazoxide Choline Controlled-Release Tablet (DCCR) in Prader–Willi Syndrome

Prader–Willi syndrome (PWS) is a complex genetic neurobehavioral/metabolic disorder with an estimated birth incidence of 1:15,000 to 1:25,000 males and females [88]. Clinical features of PWS include hypotonia and poor feeding in infancy; low muscle mass is present throughout life; the accumulation of excess body fat typically begins at approximately age 2 and continues into adulthood [89]. Ultimately, the central neurological defect of the disease results in an obsession with food, aggressive food seeking, and reduced satiety. This results in hyperphagia, and a progression to morbid obesity if the energy intake is not carefully managed [89]. Intellectual disability, growth hormone deficiency, behavioral problems, including aggressive and threatening behaviors, and neuroendocrine abnormalities are also characteristic of PWS [88]. The death rate among PWS patients is markedly elevated [90]. According to a 2014 survey of parents and caregivers of PWS patients, reducing hunger and improving food-related behaviors were the most important unmet needs in PWS that could be addressed in the development of a new therapeutic [91]. There are no approved therapeutics for the treatment of hyperphagia in PWS. DCCR is a novel, patent-protected, extended-release, crystalline salt tablet formulation of diazoxide which is administered once daily.

The effect of DCCR in PWS was tested in clinical study PC025, a single-center pilot study which enrolled 13 overweight or obese male and female subjects between the ages of 10 and 22 years old, with genetically confirmed PWS [92]. Treatment with DCCR resulted in greater improvements in hyperphagia at higher doses and in subjects with more marked baseline hyperphagia. DCCR treatment also resulted in statistically significant, dose dependent reductions in fat mass, increases in lean body mass, with a corresponding reduction in waist circumference. Treatment also resulted in the reduction of aggressive and threatening behaviors. There were trends for the improvement of lipids and insulin resistance.

## 4. Discussion

The K_ATP_ channel plays a central role in the regulation of a number of physiological processes, which, in the context of the underlying genetic or structural defects in many forms of syndromic hyperphagic obesity, cumulatively contribute to elevations in appetite and aggressive food seeking, lack of satiety, accumulation of excess body fat and the establishment and perpetuation of the obese state.

Hyperphagia, in most forms of hyperphagic obesity, is due to both enhanced orexigenic drive and diminished anorectic signaling. Activation of the K_ATP_ channel in NAG neurons should replicate the effects of leptin and insulin, hyperpolarizing the resting membrane potential of the cell and, thereby, reducing secretion of NPY and AgRP and also likely GABA. The net effect of this downregulation of secretion should be reductions in hyperphagia and more generally in appetite. These are anticipated to occur because reductions in NPY should directly and instantaneously reduce appetite. It should also result in reduced NPY-mediated suppression of the activity of prohormone convertase 2, leading to more extensive processing of POMC to αMSH and thereby enhancing anorectic signaling through the interaction of αMSH with MC4R. Reductions in AgRP, since it is an inverse agonist of MC4R, should also enhance anorectic signaling. Finally, reductions in GABA secretion by the NAG neurons should reduce the inhibitory action of GABA on POMC neurons and thereby enhance anorectic signaling. Even in the context of leptin or insulin resistance, it is possible to activate the K_ATP_ channel, resulting in the hyperpolarization of the resting membrane potential thereby reducing secretion of NPY and AgRP and, as a consequence, this approach can effectively reduce hyperphagia and appetite without a need to first restore either leptin sensitivity or insulin sensitivity. This effect on appetite would be further enhanced by improvements in satiety mediated through activation of the K_ATP_ channel in the DMV.

The accumulation of excess body fat is a fundamental characteristic of hyperphagic obesity and can be driven by excess energy intake which results in the preferential directing of excess energy to fat, or by hyperinsulinemia and/or insulin resistance. Directly activating the K_ATP_ channel in adipocytes should result in increases in β-oxidation of fat and reduced de novo synthesis of fatty acids and triglyceride accumulation. This direct effect alone should result in reduced fat deposition and/or reductions in fat mass. These effect would be complemented by the effects of K_ATP_ channel activators on hyperinsulinemia and insulin resistance. Insulin resistance tends to cause a preferential deposition of consumed energy as fat. Hyperinsulinemia has a similar effect on energy storage in fat. It also suppresses lipolysis and thereby contributes to the persistent accumulation of fat. Reductions in hyperinsulinemia result directly from the effect of activation of the K_ATP_ channel in the pancreatic β-cell, and indirectly from activating the channel in the DMV. Insulin resistance can be reduced via reductions in hyperinsulinemia and by direct effects resulting from activating the channel in the DMV. The net effect of these responses to K_ATP_ channel activation should be reduced fat mass and reduced accumulation of fat.

Beyond the effect of K_ATP_ channel activation-mediated improvements in insulin resistance on adipocytes, more generally, these improvements in insulin resistance should result in improved glycemic control.

Dyslipidemia is frequently observed in hyperphagic obesity, particularly when the individual is insulin resistant. Activating the K_ATP_ channel in the DMV has the potential to reduce hepatic synthesis and secretion of triglyceride-rich lipoprotein particles by the liver, correcting or improving dyslipidemia. Given that there is reduced triglyceride synthesis by the liver, there should, as a consequence, be reduced hepatic lipid content.

Treatment with K_ATP_ channel activators in nine animal models of hyperphagic obesity resulted in a range of therapeutic responses that are completely consistent with the predicted ressponses that follow from activating the channel in NAG neurons, the DMV, adipocytes and the pancreatic β-cell. These included reductions in hyperphagia, weight loss and body fat loss in mature animals, reductions in the rate of weight gain and body fat accumulation in growing animals, improved circulating and hepatic lipids, and improved glycemic control. These nine animal models included both genetic and induced models of hyperphagic obesity. The underlying basis for hyperphagia and excess weight gain in these models included disrupted leptin responsiveness, hyperinsulinemia, insulin resistance, and reduced satiety resulting in increased meal size.

The extant experience with DCCR in a small pilot study of patients with PWS was consistent with the animal model results, increasing the likelihood of effective translation of animal model results to clinical efficacy. In the published study, treated PWS subjects showed reductions in hyperphagia, loss of body fat, reductions in circulating lipids and improvements in insulin resistance, each anticipated from the activation of the K_ATP_ channel and consistent with the results observed in animal models of hyperphagic obesity. These results provide motivation to the more extensive evaluation of the efficacy of K_ATP_ channel activators in PWS.

Based on the animal model results, treatment of subjects who are genetically predisposed to hyperphagic obesity, but do not yet present with either hyperphagia or marked obesity, could result in preventing or delaying the transition to hyperphagia, to limiting the accumulation of excess body fat and to delaying or preventing glycemic dysregulation, which consistently follows obesity. Similarly, treatment of subjects who are obese and hyperphagic could result in the reduction of elimination of hyperphagia, reduction in body fat, improved lipid profiles and, potentially, improvements in glycemic control.

## 5. Conclusions

Given this range of relevant therapeutic responses that follow from activating the K_ATP_ channel, pharmacological activators of the channel could be a useful treatment option in syndromic hyperphagic obesity and may have utility in delaying the progression of these conditions, where obesity and hyperphagia are not evident from birth.

## Figures and Tables

**Table 1:** Tabular summary of responses to K_ATP_ channel activator treatment in nine animal models of hyperphagic obesity.

Model	Energy Intake	Weight	Body Fat	Glycemic Control	Circulating Lipids	Hepatic Lipids
Magel2 mouse	NM	Weight loss	Loss of body fat	Improved	NM	NM
ZF rat	Reduced	Reduced rate of gain	NM	Improved	Improved	NM
ZDF rat	Reduced	Reduced rate of gain	NM	Improved	Improved	Improved
db/db mouse	Reduced	NM	NM	NM	NM	NM
OTLEF rat	Reduced	Reduced rate of gain	NM	Improved	Improved	Improved
Hypothalamic injury rat	Reduced	NM	NM	NM	NM	NM
Hypothalamic injury chicken	Reduced	NM	NM	NM	NM	NM
Streptozotocin diabetic rat	NM	Reduced rate of gain	NM	NM	NM	NM
HFD obese mouse	Reduced	Weight loss or reduced rate of gain	Loss of body fat	Improved	Improved	NM

NM—parameter was not measured.

## References

[1] Dubern B., Clément K. (2012). Leptin and leptin receptor-related monogenic obesity. Biochimie.

[2] Butler M.G., Miller J.L., Forster J.L. (2019). Prader-Willi Syndrome—Clinical Genetics, Diagnosis and Treatment Approaches: An Update. Curr. Pediatr. Rev..

[3] Lustig R.H. (2008). Hypothalamic obesity: Causes, consequences, treatment. Pediatr. Endocrinol. Rev..

[4] Foster M.N., Coetzee W. (2016). KATP Channels in the Cardiovascular System. Physiol. Rev..

[5] Baquero A.F., De Solis A.J., Lindsley S.R., Kirigiti M.A., Smith M.S., Cowley M., Zeltser L., Grove K.L. (2014). Developmental switch of leptin signaling in arcuate nucleus neurons. J. Neurosci..

[6] Shi H., Moustaid-Moussa N., Wilkison W.O., Zemel M. (1999). Role of the sulfonylurea receptor in regulating human adipocyte metabolism. FASEB J..

[7] Krashes M.J., Shah B.P., Koda S., Lowell B.B. (2013). Rapid versus delayed stimulation of feeding by the endogenously released AgRP neuron mediators GABA, NPY, and AgRP. Cell Metab..

[8] Cansell C., Denis R., Joly-Amado A., Castel J., Luquet S.H. (2012). Arcuate AgRP neurons and the regulation of energy balance. Front. Endocrinol..

[9] Cyr N.E., Toorie A.M., Steger J.S., Sochat M.M., Hyner S., Perelló M., Stuart R., Nillni E.A. (2013). Mechanisms by which the orexigen NPY regulates anorexigenic α-MSH and TRH. Am. J. Physiol. Metab..

[10] Bouret S.G. (2013). Organizational actions of metabolic hormones. Front. Neuroendocr..

[11] Beck B. (2006). Neuropeptide Y in normal eating and in genetic and dietary-induced obesity. Philos. Trans. R. Soc. B Boil. Sci..

[12] Luquet S.H., Perez F.A., Hnasko T.S., Palmiter R.D. (2005). NPY/AgRP Neurons Are Essential for Feeding in Adult Mice but Can Be Ablated in Neonates. Science.

[13] Baver S.B., Hope K., Guyot S., Bjørbaek C., Kaczorowski C., O’Connell K.M.S. (2014). Leptin modulates the intrinsic excitability of AgRP/NPY neurons in the arcuate nucleus of the hypothalamus. J. Neurosci..

[14] Spanswick D., Smith M.A., Groppi V.E., Logan S.D., Ashford M. (1997). Leptin inhibits hypothalamic neurons by activation of ATP-sensitive potassium channels. Nature.

[15] Top M.V.D., Lee K., Whyment A.D., Blanks A.M., Spanswick D.C. (2004). Orexigen-sensitive NPY/AgRP pacemaker neurons in the hypothalamic arcuate nucleus. Nat. Neurosci..

[16] Ramamoorthy P., Wang Q., Whim M.D. (2011). Cell Type-Dependent Trafficking of Neuropeptide Y-Containing Dense Core Granules in CNS Neurons. J. Neurosci..

[17] Stricker-Krongrad A., Barbanel G., Beck B., Burlet A., Nicolas J., Burlet C. (1993). K+-stimulated neuropeptide Y release into the paraventricular nucleus and relation to feeding behavior in free-moving rats. Neuropeptides.

[18] Top M.V.D., Lyons D., Lee K., Coderre E., Renaud L., Spanswick D.C. (2007). Pharmacological and molecular characterization of ATP-sensitive K+ conductances in CART and NPY/AgRP expressing neurons of the hypothalamic arcuate nucleus. Neuroscience.

[19] Laughton W.B., Powley T.L. (1987). Localization of efferent function in the dorsal motor nucleus of the vagus. Am. J. Physiol. Integr. Comp. Physiol..

[20] Berthoud H.-R. (2008). The vagus nerve, food intake and obesity. Regul. Pept..

[21] Saper C.B., Loewy A., Swanson L., Cowan W. (1976). Direct hypothalamo-autonomic connections. Brain Res..

[22] Swanson L.W., Sawchenko P.E. (1980). Parventricular nucleus: A site for the integration of neuroendocrine and autonomic mechanisms. Neuroendocrinology.

[23] Zsombok A., Smith B. (2008). Plasticity of central autonomic neural circuits in diabetes. Biochim. Biophys. Acta.

[24] Berglund E.D., Liu T., Kong X., Sohn J.-W., Vong L., Deng Z., Lee C.E., Lee S., Williams K.W., Olson D.P. (2014). Melanocortin 4 receptors in autonomic neurons regulate thermogenesis and glycemia. Nat. Neurosci..

[25] Sohn J.-W., Harris L.E., Berglund E.D., Liu T., Vong L., Lowell B.B., Balthasar N., Williams K.W., Elmquist J.K. (2013). Melanocortin 4 receptors reciprocally regulate sympathetic and parasympathetic – preganglionic neurons. Cell.

[26] Hjelland I., Oveland N.P., Leversen K., Berstad A., Hausken T. (2005). Insulin-Induced Hypoglycemia Stimulates Gastric Vagal Activity and Motor Function without Increasing Cardiac Vagal Activity. Digestion.

[27] Maeda C.Y., Fernandes T.G., Lulhier F., Irigoyen M.C.C. (1995). Streptozotocin diabetes modifies arterial pressure and baroreflex sensitivity in rats. Braz. J. Med. Biol. Res..

[28] Plum L., Schubert M., Brüning J.C. (2005). The role of insulin receptor signaling in the brain. Trends Endocrinol. Metab..

[29] Plum L., Belgardt B.F., Brüning J.C. (2006). Central insulin action in energy and glucose homeostasis. J. Clin. Investig..

[30] Lam T.K.T., Pocai A., Gutierrez-Juarez R., Obici S., Bryan J., Aguilar-Bryan L., Schwartz G.J., Rossetti L. (2005). Hypothalamic sensing of circulating fatty acids is required for glucose homeostasis. Nat. Med..

[31] Obici S., Feng Z., Karkanias G., Baskin D.G., Rossetti L. (2002). Decreasing hypothalamic insulin receptors causes hyperphagia and insulin resistance in rats. Nat. Neurosci..

[32] Obici S., Zhang B.B., Karkanias G., Rossetti L. (2002). Hypothalamic insulin signaling is required for inhibition of glucose production. Nat. Med..

[33] Huang H.-N., Lu P.-J., Lo W.-C., Lin C.-H., Hsiao M., Tseng C.J. (2004). In Situ Akt Phosphorylation in the Nucleus Tractus Solitarii Is Involved in Central Control of Blood Pressure and Heart Rate. Circulation.

[34] Krowicki Z., Nathan N.A., Hornby P.J. (1998). Gastric motor and cardiovascular effects of insulin in dorsal vagal complex of the rat. Am. J. Physiol. Content.

[35] Blake C.B., Smith B. (2012). Insulin reduces excitation in gastric-related neurons of the dorsal motor nucleus of the vagus. Am. J. Physiol. Integr. Comp. Physiol..

[36] Pocai A., Lam T.K.T., Gutierrez-Juarez R., Obici S., Schwartz G.J., Bryan J., Aguilar-Bryan L., Rossetti L. (2005). Hypothalamic KATP channels control hepatic glucose production. Nature.

[37] Grill H.J., Schwartz M.W., Kaplan J.M., Foxhall J.S., Breininger J., Baskin D.G. (2002). Evidence that the caudal brainstem is a target for the inhibitory effect of leptin on food intake. Endocrinology.

[38] Williams K.W., Zsombok A., Smith B. (2006). Rapid inhibition of neurons in the dorsal motor nucleus of the vagus by leptin. Endocrinology.

[39] Rogers R.C., Hermann G.E. (1987). Oxytocin, oxytocin antagonist, TRH, and hypothalamic paraventricular nucleus stimulation effects on gastric motility. Peptides.

[40] Abrahamsson H., Jansson G. (1969). Elicitation of Reflex Vagal Relaxation of the Stomach from Pharynx and Esophagus in the Cat. Acta Physiol. Scand..

[41] Ferreira M., Browning K.N., Sahibzada N., Verbalis J.G., Gillis R.A., Travagli R.A. (2001). Glucose effects on gastric motility and tone evoked from the rat dorsal vagal complex. J. Physiol..

[42] Schwartz M.W., Porte D. (2005). Diabetes, Obesity, and the Brain. Science.

[43] Bogacka I., Roane D.S., Xi X., Zhou J., Li B., Ryan D., Martin R.J. (2004). Expression Levels of Genes Likely Involved in Glucose-sensing in the Obese Zucker Rat Brain. Nutr. Neurosci..

[44] Alemzadeh R., Karlstad M., Tushaus K., Buchholz M. (2008). Diazoxide enhances basal metabolic rate and fat oxidation in obese Zucker rats. Metabolism.

[45] Alemzadeh R., Tushaus K. (2005). Diazoxide attenuates insulin secretion and hepatic lipogenesis in zucker diabetic fatty rats. Med. Sci. Monit..

[46] Komatsu M., Takei M., Ishii H., Sato Y. (2013). Glucose-stimulated insulin secretion: A newer perspective. J. Diabetes Investig..

[47] Page M.M., Johnson J.D. (2018). Mild Suppression of Hyperinsulinemia to Treat Obesity and Insulin Resistance. Trends Endocrinol. Metab..

[48] Templeman N.M., Skovsø S., Page M.M., E Lim G., Johnson J.D. (2017). A causal role for hyperinsulinemia in obesity. J. Endocrinol..

[49] Gagnon A., Sorisky A. (1998). The effect of glucose concentration on insulin-induced 3T3-L1 adipose cell differentiation. Obes. Res..

[50] Nielsen T.S., Jessen N., Jørgensen J.O.L., Moller N., Lund S. (2014). Dissecting adipose tissue lipolysis: Molecular regulation and implications for metabolic disease. J. Mol. Endocrinol..

[51] Czech M.P., Tencerova M., Pedersen D.J., Aouadi M. (2013). Insulin signalling mechanisms for triacylglycerol storage. Diabetologia.

[52] Cristancho A.G., Lazar M.A. (2011). Forming functional fat: A growing understanding of adipocyte differentiation. Nat. Rev. Mol. Cell Boil..

[53] Corkey B. (2011). Banting Lecture 2011: Hyperinsulinemia: Cause or Consequence?. Diabetes.

[54] Mehran A.E., Templeman N.M., Brigidi G.S., Lim G.E., Chu K.-Y., Hu X., Botezelli J.D., Asadi A., Hoffman B., Kieffer T.J. (2012). Hyperinsulinemia Drives Diet-Induced Obesity Independently of Brain Insulin Production. Cell Metab..

[55] D’Souza A.M., Johnson J.D., Clee S.M., Kieffer T.J. (2016). Suppressing hyperinsulinemia prevents obesity but causes rapid onset of diabetes in leptin-deficient Lepob/ob mice. Mol. Metab..

[56] Wang B., Charukeshi C.P., Pippin J.J. (2014). Leptin- and Leptin Receptor-Deficient Rodent Models: Relevance for Human Type 2 Diabetes. Curr. Diabetes Rev..

[57] Gray S.L., Donald C., Jetha A., Covey S.D., Kieffer T.J. (2010). Hyperinsulinemia Precedes Insulin Resistance in Mice Lacking Pancreatic β-Cell Leptin Signaling. Endocrinology.

[58] Hales C., Kennedy G., Byrne C.D., Brindle N.P.J., Wang T.W.M., Zorzano A., Balon T.W., Brady L.J., Rivera P., Garetto L.P. (1964). Plasma glucose, non-esterified fatty acid and insulin concentrations in hypothalamic-hyperphagic rats. Biochem. J..

[59] York D.A., Bray G.A. (1972). Dependence of Hypothalamic Obesity on Insulin, the Pituitary and the Adrenal Gland11. Endocrinology.

[60] Han P., Frohman L. (1970). Hyperinsulinemia in tube-fed hypophysectomized rats bearing hypothalamic lesions. Am. J. Physiol. Content.

[61] Goldman J., Bernardis L., Frohman L. (1974). Food intake in hypothalamic obesity. Am. J. Physiol. Content.

[62] Kobayashi M., Olefsky J.M. (1978). Effect of experimental hyperinsulinemia on insulin binding and glucose transport in isolated rat adipocytes. Am. J. Physiol. Metab..

[63] Rizza R.A., Mandarino L.J., Genest J., Baker B.A., Gerich J.E. (1985). Production of insulin resistance by hyperinsulinemia in man. Diabetologia.

[64] Shanik M.H., Xu Y., Škrha J., Dankner R., Zick Y., Roth J. (2008). Insulin Resistance and Hyperinsulinemia: Is hyperinsulinemia the cart or the horse?. Diabetes Care.

[65] Ratzmann K.P., Ruhnke R., Kohnert K.D. (1983). Effect of pharmacological suppression of insulin secretion on tissue sensitivity to insulin in subjects with moderate obesity. Int. J. Obes..

[66] Wigand J., Blackard W. (1979). Downregulation of insulin receptors in obese men. Diabetes.

[67] Alemzadeh R., Zhang J., Tushaus K., Koontz J. (2004). Diazoxide enhances adipose tissue protein kinase B activation and glucose transporter-4 expression in obese Zucker rats. Med. Sci. Monit..

[68] Alemzadeh R., Tushaus K.M. (2004). Modulation of Adipoinsular Axis in Prediabetic Zucker Diabetic Fatty Rats by Diazoxide. Endocrinology.

[69] Škrha J., Svacina S., Šrámková J., Páv J. (1989). Use of euglycaemic clamping in evaluation of diazoxide treatment of insulinoma. Eur. J. Clin. Pharmacol..

[70] Lutz T.A., Woods S.C. (2012). Overview of animal models of obesity. Curr. Protoc. Pharmacol..

[71] Dube M.G., Kalra S.P., Kalra P.S. (2007). Low abundance of NPY in the hypothalamus can produce hyperphagia and obesity. Peptides.

[72] Bischof J.M., Wevrick R. (2018). Chronic diazoxide treatment decreases fat mass and improves endurance capacity in an obese mouse model of Prader-Willi syndrome. Mol. Genet. Metab..

[73] Knani I., Earley B.J., Udi S., Nemirovski A., Hadar R., Gammal A., Cinar R., Hirsch H.J., Pollak Y., Gross I. (2016). Targeting the endocannabinoid/CB1 receptor system for treating obesity in Prader-Willi syndrome. Mol. Metab..

[74] Dryden S., Pickavance L., Frankish H.M., Williams G. (1995). Increased neuropeptide Y secretion in the hypothalamic paraventricular nucleus of obese (fa/fa) Zucker rats. Brain Res..

[75] Alemzadeh R., Slonim A.E., Zdanowicz M.M., Maturo J. (1993). Modification of insulin resistance by diazoxide in obese Zucker rats. Endocrinology.

[76] Alemzadeh R., Jacobs W., Pitukcheewanont P. (1996). Antiobesity effect of diazoxide in obese zucker rats. Metabolism.

[77] Alemzadeh R., Holshouser S. (1999). Effect of diazoxide on brain capillary insulin receptor binding and food intake in hyperphagic obese Zucker rats. Endocrinology.

[78] Standridge M., Alemzadeh R., Zemel M., Koontz J., Moustaid-Moussa N. (2000). Diazoxide down-regulates leptin and lipid metabolizing enzymes in adipose tissue of Zucker rats. FASEB J..

[79] Hensley I., E Lawler J., Alemzadeh R., Holshouser S.J. (2001). Diazoxide effects on hypothalamic and extra-hypothalamic NPY content in Zucker rats. Peptides.

[80] De Luca C., Kowalski T.J., Zhang Y., Elmquist J.K., Lee C., Kilimann M.W., Ludwig T., Liu S.-M., Chua S.C. (2005). Complete rescue of obesity, diabetes, and infertility in db/db mice by neuron-specific LEPR-B transgenes. J. Clin. Investig..

[81] Lee S. (1981). Effects of diazoxide on insulin secretion and metabolic efficiency in the db/db mouse. Life Sci..

[82] Bi S., Moran T.H. (2016). Obesity in the Otsuka Long Evans Tokushima Fatty Rat: Mechanisms and Discoveries. Front. Nutr..

[83] Guo Z., Bu S., Yu Y., Ghatnekar G., Wang M., Chen L., Bu M., Yang L., Zhu B., Feng Z. (2008). Diazoxide prevents abdominal adiposity and fatty liver in obese OLETF rats at prediabetic stage. J. Diabetes Complicat..

[84] Larue-Achagiotis C., Le Magnen J. (1978). Effects of a Diazoxide inhibition of insulin release on food intake of normal and hyperphagic hypothalamic rats. Pharmacol. Biochem. Behav..

[85] Sonoda T. (1983). Hyperinsulinemia and its role in maintaining the hypothalamic hyperphagia in chickens. Physiol. Behav..

[86] Matsuda M., Kawasaki F., Mikami Y., Takeuchi Y., Saito M., Eto M., Kaku K. (2002). Rescue of beta-cell exhaustion by diazoxide after the development of diabetes mellitus in rats with streptozotocin-induced diabetes. Eur. J. Pharmacol..

[87] Surwit R.S., Dixon T.M., Petro A.E., Daniel K.W., Collins S. (2000). Diazoxide restores β3-adrenergic receptor function in diet-induced obesity and diabetes. Endocrinology.

[88] McCandless S.E., Committee on Genetics American Academy of Pediatrics (2011). Clinical Report-Health supervision for children with Prader-Willi syndrome. Pediatrics.

[89] Miller J.L., Lynn C.H., Driscoll D.C., Goldstone A.P., Gold J.-A., Kimonis V., Dykens E., Butler M.G., Shuster J.J., Driscoll D.J. (2011). Nutritional phases in Prader-Willi syndrome. Am. J. Med. Genet. Part. A.

[90] Hedgeman E., Ulrichsen S.P., Carter S., Kreher N.C., Malobisky K.P., Braun M.M., Fryzek J., Olsen M.S. (2017). Long-term health outcomes in patients with Prader–Willi Syndrome: A nationwide cohort study in Denmark. Int. J. Obes..

[91] Summary of the Impact of PWS on Individuals and Their Families and Views on Treatments: Results of an International Online Survey. https://www.fpwr.org/pws-patient-voices.

[92] Kimonis V.E., Surampalli A., Wencel M., Gold J.-A., Cowen N.M. (2019). A randomized pilot efficacy and safety trial of diazoxide choline controlled-release in patients with Prader-Willi syndrome. PLoS ONE.

